# The clinical characteristics and pathogenic variants of primary pigmented nodular adrenocortical disease in 210 patients: a systematic review

**DOI:** 10.3389/fendo.2024.1356870

**Published:** 2024-06-26

**Authors:** Julian Sun, Lin Ding, Liping He, Hang Fu, Rui Li, Jing Feng, Jianjun Dong, Lin Liao

**Affiliations:** ^1^ School of Clinical Medicine, Shandong Second Medical University, Weifang, China; ^2^ Department of Endocrinology and Metabology, The First Affiliated Hospital of Shandong First Medical University & Shandong Provincial Qianfoshan Hospital, Jinan, China; ^3^ Department of Endocrinology and Metabology, Shandong Provincial Qianfoshan Hospital, Jinan, China; ^4^ First Clinical Medical College, Shandong University of Traditional Chinese Medicine, Jinan, China; ^5^ Department of Endocrinology, Qilu Hospital of Shandong University, Jinan, China; ^6^ Division of Endocrinology, Department of Internal Medicine, Qilu Hospital of Shandong University, Jinan, China

**Keywords:** *PRKAR1A*, Cushing’s syndrome, clinical characteristics, pathogenic variants, primary pigmented nodular adrenocortical disease

## Abstract

**Aims:**

Primary pigmented nodular adrenocortical disease (PPNAD), as a rare kind of Cushing’s syndrome, is frequently misdiagnosed. To get a better understanding of the disease, we analyzed the clinical characteristics and pathogenic variants of PPNAD.

**Methods:**

Databases were searched, and the pathogenic variants and clinical manifestations of patients were summarized from the relevant articles.

**Results:**

A total of 210 patients in 86 articles were enrolled with a median age of 22 and a female-to-male ratio of 2:1. Sixty-six (31.43%) patients were combined with Carney complex (CNC) and 94.29% were combined with osteoporosis/osteopenia. Among 151 patients who underwent genetic testing, 87.42% (132/151) had pathogenic variants. Six gene mutations (*PRKAR1A*, *PDE11A*, *PRKACA*, *CTNNB1*, *PDE8B*, and *ARMC5*) were detected in the patients. The most common mutation was PKAR1A, accounting for 79.47% (120/151). There was a significant correlation between *PRKAR1A* pathogenic variant and spotty skin pigmentation in CNC concurrent with PPNAD (*p* < 0.05). Among pregnant patients with PPNAD, those without surgical treatment and with bilateral adrenalectomy suffered from a high-risk perinatal period. However, patients with unilateral adrenalectomy presented a safe perinatal period.

**Conclusions:**

For young patients with Cushing’s syndrome, especially female patients with spotty skin pigmentation and osteoporosis/osteopenia, PPNAD should be considered. Unilateral adrenal resection may be considered as an option for women with fertility needs. In view of the difficulty of PPNAD diagnosis, genetic testing before surgery might be a reasonable option. Patients with PPNAD with spotty skin pigmentation should consider the *PRKAR1A* pathogenic variant and pay attention to CNC.

**Systematic review registration:**

https://www.crd.york.ac.uk/prospero, identifier CRD42023416988.

## 1 Introduction

Primary pigmented nodular adrenocortical disease (PPNAD), as a subclass of bilateral micronodular adrenocortical disease, is a rare but an important cause of endogenous Cushing’s syndrome (CS) especially in children and young adults. Characterized by small, black and brown pigmented micronodules in the adrenal cortex, PPNAD can be combined with the “complex of myxomas, spotty skin pigmentation, and endocrine overactivity,” or Carney complex (CNC) (cPPNAD) (1). Meanwhile, 10%–20% of patients with PPNAD can also occur in patients without CNC [denoted as isolated PPNAD (i-PPNAD)] (2). Pathologically, the diagnosis of PPNAD is mainly based on histological findings, which displays multiple adrenocortical nodules with cytoplasmic pigmentation and inter-nodular cortical atrophy. However, radiology may underestimate the presence of bilateral adrenal gland involvement in patients with PPNAD due to the discrete nodular formations (sizes < 1 cm). These micronodules are often cortisol-producing and composed of lipid-poor cortical cells; therefore, hypercortisolism in PPNAD can be overt, subclinical, cyclic, or atypical (3, 4). All these make the diagnosis of PPNAD difficult. In addition, cPPNAD is causally related with inactivating mutations of the regulatory subunit type 1A of the cAMP-dependent protein kinase (*PRKAR1A*) gene and yet unknown defect(s) in other gene(s). PPNAD is a rare autosomal dominant disorder with variable penetrance and uncertain genotype/phenotype correlation. Delineation of a genotype–phenotype correlation for patients with PPNAD is essential for understanding potential gene functions and providing counseling and preventive care. In this review, we summarize the clinical features, pathogenic variants, and recent progress in investigation and therapy of PPNAD.

## 2 Subjects and methods

### 2.1 Data sources and study patients

**Figure 1:**
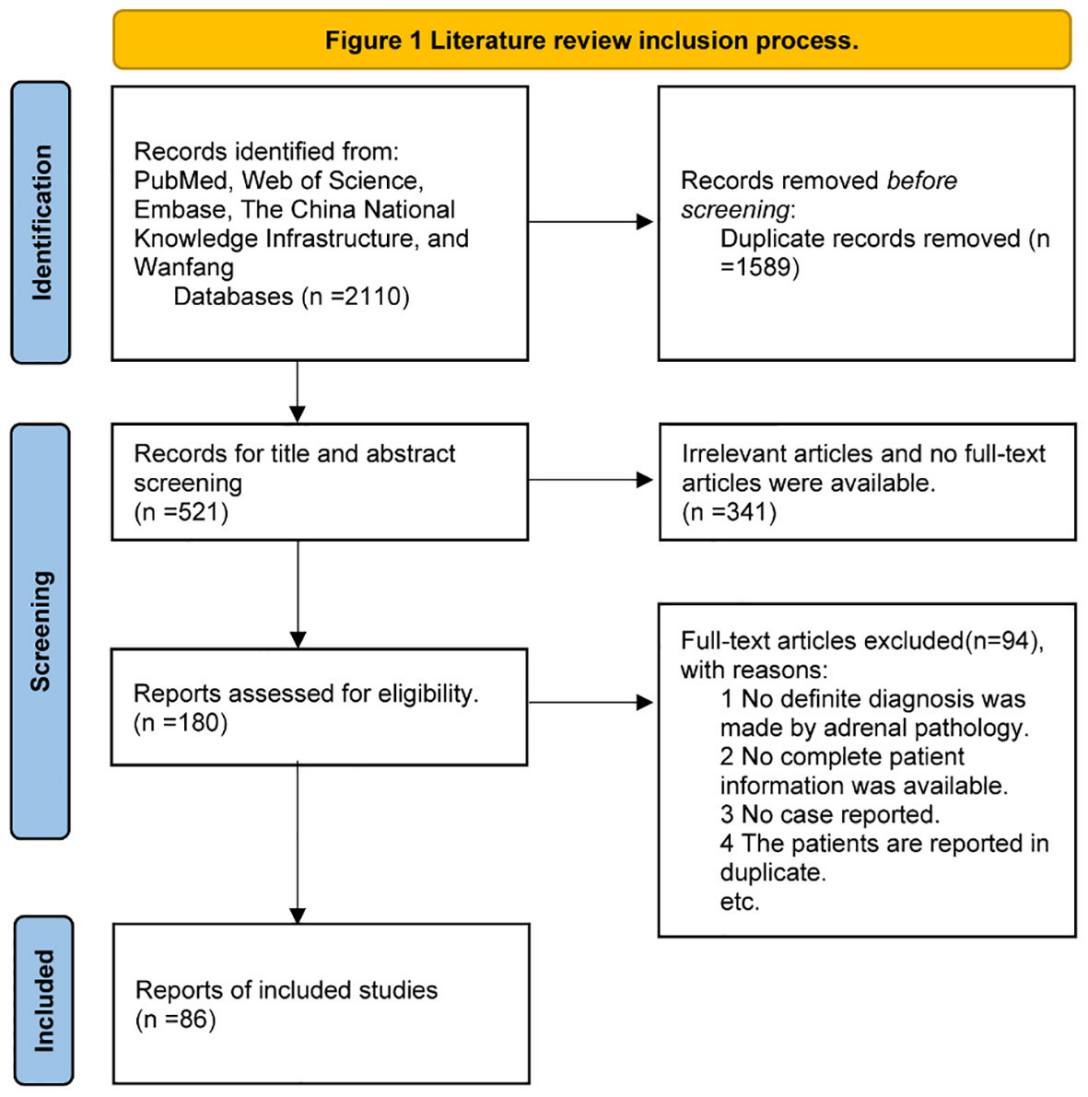
Literature review inclusion process.

Five electronic databases (i.e., PubMed, Web of Science, Embase, the China National Knowledge Infrastructure, and Wanfang) were used to search for relevant studies with the following terms: “PPNAD” or “iPPNAD” or “familial isolated primary pigmented nodular adrenocortical disease” or “isolated primary pigmented nodular adrenocortical disease” or “primary pigmented nodular adrenocortical disease” or “micronodular adrenal disease”. Analyses of pathogenic variants and clinical features were conducted from inception to 12 September 2023. We selected studies in the English or Chinese language. Eligible studies met the following criteria: (1) PPNAD diagnosed by pathology after adrenalectomy according to the 2022 WHO classification of adrenal cortical tumors {PPNAD is composed of multiple beaded pigmented micronodules (<10 mm, often 2–5 mm). PPNAD is characterized by multiple subcentimeter micronodules composed of eosinophilic adrenocortical cells with different pigment deposits. Atrophy of the intertubercular cortex is commonly observed. Micronodules were positive for CYP11B1, confirming cortisol production (5)}. (2) The pathogenic variant sites were described. (3) The main clinical data of the patients were described. The flow diagram of the search process is provided in **Figure 1**.

The following data were extracted from the eligible studies: (1) country, (2) sex, (3) age at diagnosis of PPNAD, (4) pathogenic variant, (5) treatment, (6) clinical features, and (7) complication of CNC. Diagnostic criteria of CNC are shown in **Table 1**. The CNC diagnostic criteria were based on the criteria proposed by Stratakis et al. (1).

**Table 1:** Diagnostic criteria for CNC^*^.

1. Spotty skin pigmentation with a typical distribution (lips, conjunctiva and inner or outer canthi, and vaginal and penile mucosa)
2. Myxoma (cutaneous and mucosal) ^#^
3. Cardiac myxoma^#^
4. Breast myxomatosis^#^ or fat-suppressed magnetic resonance imaging findings suggestive of this diagnosis
5. PPNAD^#^ or paradoxical positive response of urinary glucocorticosteroids to dexamethasone administration during Liddle’s test
6. Acromegaly due to GH-producing adenoma^#^
7. LCCSCT^#^ or characteristic calcification on testicular ultrasonography
8. Thyroid carcinoma^#^ or multiple, hypoechoic nodules on thyroid ultrasonography, in a young patient
9. Psammomatous melanotic schwannoma^#^
10. Blue nevus, epithelioid blue nevus (multiple)^#^
11. Breast ductal adenoma (multiple)^#^
12. Osteochondromyxoma^#^
*Supplemental criteria:*
1. Affected first-degree relative
2. Inactivating mutation of the PRKAR1A gene

*To make a diagnosis of CNC, a patient must either (1) exhibit two of the manifestations of the disease listed or (2) exhibit one of these manifestations and meet one of the supplemental criteria (an affected first-degree relative or an inactivating mutation of the PRKAR1A gene).

^#^With histologic confirmation.

Liddle GW was the first person using dexamethasone to assess CS in 1960. The original low-dose dexamethasone suppression test (LDDST) and high-dose dexamethasone suppression test (HDDST) were named Liddle tests, which were widely used in the evaluation of CS (6).

### 2.2 Statistical analysis

The epidemiological and clinical characteristics, and laboratory indexes of patients were described utilizing simple summary statistics. Fisher exact tests were used to test for association between qualitative variables. All tests were two-sided, and *p*-values < 0.05 were considered statistically significant. Statistical analysis was performed using the Statistical Package for the Social Sciences version 26 for Windows (SPSS). Since certain data in some patients were missing, the total number of patients was mentioned in each analysis.

## 3 Results

### 3.1 Epidemiological characteristics

**Figure 2:**
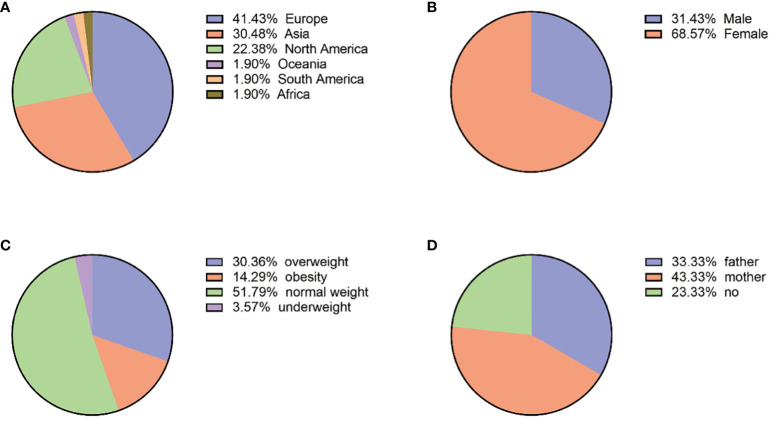
**(A)** Geographical country distribution. **(B)** Gender distribution. **(C)** BMI (N:56). **(D)** Mutated genes from parents (N:30).

Eighty-six articles including 210 patients were enrolled. The top three countries were France (65/210, 30.95%), China (43/210, 20.48%), and Canada (27/210, 12.86%). The patients were distributed in 23 countries on six continents. Europe has the highest number of cases (87/210, 41.43%), followed by Asia (64/210, 30.48%), North America (47/210, 22.38%), Africa and Oceania (8/210, 3.81%), and South America (4/210, 1.90%) (**Table 2**; **Figure 2**). A total of 26 patients were from 11 families, and the others were considered as sporadic.

**Table 2:** The detailed information of patients with PPNAD.

Continent	Percentage	Patients (%)
Europe	French	65 (30.95)
	Belgium	7 (3.33)
	Poland	5 (2.38)
	Portugal	3 (1.43)
	Greece	2 (0.95)
	The United Kingdom	2 (0.95)
	Germany	1 (0.48)
	Serbia	1 (0.48)
	Austria	1 (0.48)
Asia	China	43 (20.48)
	Japan	6 (2.86)
	India	4 (1.90)
	Thailand	3 (1.43)
	Turkey	3 (1.43)
	Korea	2 (0.95)
	Iran	2 (0.95)
	Israel	1 (0.48)
North America	Canada	27 (12.86)
	The United States	20 (9.52)
Oceania	Australia	4 (1.90)
South America	Brazil	4 (1.90)
Africa	*	3 (1.43)
	Ethiopia	1 (0.48)
Total	23	210 (100)

*People of African descent.

### 3.2 Clinical features

The ages of patients ranged from 1 to 61 years old, with a median of 22 (quartiles 14–28), and 71.88% (151/210) of patients were 10–30 years old at diagnosis of PPNAD. Among them, female patients were 144 (144/210, 68.57%), with a female-to-male ratio of 2:1.

The clinical data are shown in **Figure 2**. In addition, BMI at diagnosis was only available for 56 patients, among which the prevalence of obesity (BMI ≥ 30 kg/m^2^) was 14.29% (8/56), overweight (BMI: 25–29.9 kg/m^2^) was 30.36% (17/56), and normal weight was 51.79% (29/56) with a median of 24.45 (quartiles 22.29–26.48) kg/m^2^. In addition, 28 patients had growth retardation, aged 1 to 19 years old with a median of 11 (quartiles 9–12). Clinical manifestations were not available in all patients, which included mainly osteoporosis or low bone mineral density (33/35, 94.29%), hypertension (81/120, 67.50%), and weight gain (71/120, 59.17%).

A total of 66 (66/210, 31.43%) patients were cPPNAD and had details of the diagnosis of CNC, with 40 (40/66, 60.61%) being women, 47 (47/66, 71.21%) having spotty skin pigmentation with a typical distribution, 19 (19/66, 28.79%) having cutaneous or cardiac myxomas, and 7 (7/40, 17.50%) having multiple breast nodules or carcinoma. Particularly, one patient with PPNAD only showed spotty pigmentation of the skin, with suspected CNC (7).

### 3.3 Laboratory investigations

ACTH data and normal criteria were available in 98 patients, 77 (77/98, 78.57%) of whom had low or undetectable ACTH, and 21 (21/98, 21.43%) were within the lower limit of the normal range of ACTH levels.

Seventy-one patients mentioned diurnal cortisol changes, among which 70 (70/71, 98.59%) lost their cortisol circadian rhythm. Moreover, regarding the dexamethasone suppression test, 31 patients were tested for urinary cortisol after high-dose dexamethasone test or Liddle test, and all (31/31, 100%) of them showed no significant suppression or even paradoxical increase. Plasma cortisol showed similar results and was not suppressed in all the patients with low-dose dexamethasone and high-dose dexamethasone inhibition tests.

Plasma dehydroepiandrosterone sulfate (DHEA-S) was available in 28 patients, normal in 11 (11/28, 39.29%) patients, decreased in 14 (14/28, 50.00%), and elevated in 3 (3/28, 10.71%) patients. Plasma growth hormone (GH) was provided in 13 patients, which was normal in 7 (7/13, 53.85%), decreased in 2 (2/13, 15.38%), and elevated in 4 (4/13, 30.77%) patients.

### 3.4 Pathogenic variants

A total of 151 patients underwent genetic testing, and 132 (132/151, 87.42%) patients found pathogenic variants. Six different mutations, *PRKAR1A*, *PDE11A*, *PRKACA*, *CTNNB1*(*β- catenin*), *PDE8B*, and *ARMC5*, were identified. The most common mutation was *PKAR1A*, accounting for 79.47% (120/151) of cases; the second most common was *PED11A* mutation, found in 26.49% (40/151) of cases, while other mutations were relatively rare. A total of 33 patients had both *PRKAR1A* and *PDE11A* mutations (**Tables 3**, **4**).

**Table 3:** Geographic distribution of genes.

Gene (patients)	Continent	Patients (%)
*PRKAR1A* (120)	Europe	75 (62.50%)
	North America	24 (20.00%)
	Asia	15 (12.50%)
	Africa	4 (3.33%)
	South America	2 (1.67%)
*PDE11A* (40)	Europe	32 (80%)
	North America	6 (15%)
	Asia	2 (5%)
*PDE8B* (2)	Asia	1 (50%)
	North America	1 (50%)
*PRKACA* (2)	Asia	2 (100%)
*CRNNB1*(2)	North America	2 (100%)
*ARMC* (1)	Asia	1 (100%)

**Table 4:** Mutations in patients with PPNAD.

No.	Gene	Region	Mutation	Amino acid	Mutation type	Somatic/Germline	Author
1	*PRKACA*	chr19p13.2p12			Copy duplication	Somatic	Xu Yuying (8), 2023
2	*PRKACA*		c.95A>T				Wan Shuang (9), 2022
3	*PRKAR1A*		c.709-16(IVS7)_c.709-11(IVS7)del TTATTT				
4	*PRKAR1A*		c.491_492delTG	p.Val164fsX4		Germline	Yuya Tsurutani (10), 2022
5	*PDE11A*	Exon 11	c.1921A>G	p.Lys641Glu	Missense		Qian Sun (11), 2022
6	*PRKAR1A*	Exon 6	c.543_544del	p.Glu181Aspfs*6	Protein-truncating		Sofia H. Ferreira (12), 2019
8	*PRKAR1A*		c.487–488delAC				Andreas Kiriakopoulos (13), 2018
9	*PRKAR1A*	Intron 3	c.349−5_349−4insT				J. Fu (14), 2018
		Intron 4a	c.440 + 5 G>C		Deletion of exon 4a		
		Intron 6	c.708 + 134_708 + 135insCT				
		Intron 7	c.770−24G>A				
		Intron 8	c.892–34G>T				
10	*PRKAR1A*	Exon 5	c.502G > A	p.168Gly>Ser			Lan Ling (15), 2017
	*PDE11A*	Exon 20	c.2763_2764insTCC	p.Ser921_Pro922insSer			
11	*PRKAR1A*		c.671delG	p.G225Afs*16	Frame shift		Laura C. Hern´andez-Ram´ırez (16), 2017
12	*PRKAR1A*		del184–237				Delphine Vezzosi (17), 2015
13	*PRKAR1A*		c.709 (-8–3)delATTTTT				
14	*PRKAR1A*		c.502 + 1G>A				
15	*PRKAR1A*		c.63_64CG>GA				
16	*PRKAR1A*		c.46C>T	p.R16X		Germline	Shih-Chen Tung (18), 2012
17	*PRKAR1A*	Exon 2	c.95_96delAA	p.Lys32Argfs*12	Frame shift		Emilie Morin (19), 2012
18	*PRKAR1A*	Intron 3	c.349 G>T		Frame shift		Helen L. Storr (20), 2010
19	*PRKAR1A*	Exon 2	(A29A)CGG→GCA				Christian Urban (21), 2007
		Exon 3	c.286C>T	R96X	Nonsense		
		Intron 7	c.769 + 524G>T				
		Intron 7	c.770–24A>G				
		Intron 9	c.974–102A>T				
20	*PRKAR1A*		c.682C>T	p.Arg228Ter		Germline	Crystal D. C. Kamilaris (22), 2021
			c.974–2A>G				
21	*PRKAR1A*		c.488delC	p.Thr163MetfsX2	Frame shift		Catherine D Zhang (23), 2019
22	*PRKAR1A*		c.709 (-7–2) del6				Aglaia Kyrilli (24), 2019
23	*PRKAR1A*		c.46C>T	p.Arg16X			Amit Tirosh (25), 2017
24	*PRKAR1A*		c.101_105delCTATT	p.Ser34fsX9			
25	*PRKAR1A*		c.496C>T	p.Gln166X			
26	*PRKAR1A*		c. 491–492delTG	p.Val164AspfsX5			
27	*PRKAR1A*		c.125dupG				Katarzyna Pasternak-Pietrzak (26), 2018
28	*PRKAR1A*		c.15dupT				
29	*PRKAR1A*	Intron 7	c.709–7_709–2del6				Constanza Navarro Moreno (3), 2018
30	*PRKAR1A*		c.1A>G	p.Met1Val			
31	*PRKAR1A*	Intron 7	c.709–7_709–2del6				
32	*PRKAR1A*		g.114213T>G				Sira Korpaisarn (27), 2017
			c.709–5T>G				
34	*PRKAR1A*		c.49G>T	p.E17X			Ryohei Mineo (28), 2016
35	*PRKAR1A*	Exon 3	243–252ACTCGTAGAGdel		Frame shift	Germline	R. M. G. da Silva (29), 2011
36	*PRKAR1A*	Exon 8	c.709(-7–2)del6 deletion				Thekla Poukoulidou (30), 2014
37	*PRKAR1A*		Q28X		Protein-truncating		Johannes Hofland (31), 2013
38	*PRKAR1A*		c.439A>G	p.S147G		Germline	J. Anselmo (32), 2012
39	*PRKAR1A*	Intron 2	c.177 + 3 A>G				Marcia C. Peck (33), 2010
40	*PRKAR1A*		c.693insT	p.Arg232X		Germline	Mimi Tadjine (34), 2008
41	*PRKAR1A*		c.1 A > G	p.Met1Val		Germline	
42	*PRKAR1A*		c.101–105delCTATT	p.Ser34fsX9		Germline	
43	*PDE11A*		171delTfs41X			Germline	
44	*PRKAR1A*		c.891 + 3 A > G			Germline	
45	*PRKAR1A*		c.438 A > T	p.Arg146Ser		Germline	
46	*PRKAR1A*		c.682C > T	p.Arg228X		Germline	
47	*PRKAR1A*		c.709-(5–107) del103			Germline	
	*CRNNB1*		T41A			Somatic	
48	*CRNNB1*		S45P			Somatic	
49	*PRKAR1A*		c.502 + 5delG			Germline	
50	*PRKAR1A*		c.491–492delTG	p.Val164fsX4		Germline	
51	*PRKAR1A*		IVS1–2 A>G				Paul Byron Bandelin (35), 2008
52	*PRKAR1A*	Exon 2		p. Y21X	Codon stop	Germline	Madson Q. Almeida (36), 2008
53	*PDE8B*		c.914A>C	p.His305Pro			Anelia Horvath (37), 2008
54	*PDE11A*			R804H			Anelia Horvath (38), 2006
55	*PRKAR1A*	Exon 8	IVS+3G>A				Isabelle Bourdeau (39), 2003
56	*PRKAR1A*		88A>G				
57	*PRKAR1A*		211 C>T				
58	*PRKAR1A*	Exon 1B	102 G>A		Cryptic splice site, partial exon skipping	Germline	Lionel Groussin (40), 2002
		Exon 4B	IVS del (-17→-2)		Exon skipping		
59	*PRKAR1A*	Exon 8	933 ins A		Frame shift	Germline	
60	*PRKAR1A*	Exon 2	196 C>T		Stop codon	Germline	
61	*PRKAR1A*	Exon 7	IVS del (-7→-2)		Exon skipping	Germline	
62	*PRKAR1A*	Exon 2	172 del 11 bp		Frame shift	Germline	
63	*PRKAR1A*	Exon 5	c.503G>T	p.Gly168Val		Germline	Haremaru Kubo (41), 2022
64	*PDE11A*		(919C)→(R307X)				J. Aidan Carney (42), 2010
65	*PDE11A*		1655_1657delTCT/insCCfs15X				
66	*PRKAR1A*		c.974–1G>A				Rossella Libè (43), 2010
	*PDE11A*		c.2618T>C	I873T	Missense		
67	*PRKAR1A*		c.502 + 1G>A				
	*PDE11A*		c.2599C>G	R867G	Missense		
68	*PRKAR1A*		c.1083delA				
	*PDE11A*		c.2180A>G	Y727C	Missense		
69	*PRKAR1A*		c.491–492delTG				
	*PDE11A*		c.171Tdel	T58PfsX41	Codon stop		
70	*PRKAR1A*		c.891 + 3A>G				
	*PDE11A*		c.2599C>G	R867G	Missense		
71	*PRKAR1A*		c.528–531delGATTins11				
	*PDE11A*		c.2632A>G	M878V	Missense		
72	*PRKAR1A*		c.682C>T				
	*PDE11A*		c.2599C>G	R867G	Missense		
73	*PRKAR1A*		c.769 + 5G>C				
	*PDE11A*		c.1045G>A	A349T	Missense		
74	*PRKAR1A*		c.709(-7–2)del6				
	*PDE11A*		c.919C>T	R307X	Codon stop		
75	*PRKAR1A*		c.709(-7–2)del6				
	*PDE11A*		c.2411G>A	R804H	Missense		
76	*PRKAR1A*		c.709(-7–2)del6				
	*PDE11A*		c.2180A>G	Y727C	Missense		
77	*PRKAR1A*		c.891 + 3A>G				
	*PDE11A*		c.2632A>G	M878V	Missense		
78	*PRKAR1A*		c.763–764delAT				
	*PDE11A*		c.2180A>G	Y727C	Missense		
79	*PRKAR1A*		c.550(-9–2)del8				
	*PDE11A*		c.2411G>A	R804H	Missense		
80	*PRKAR1A*		c.845–846 ins A				
	*PDE11A*		c.2411G>A	R804H	Missense		
81	*PRKAR1A*		c.709(-5–107)del 103				
	*PDE11A*		c.2180A>G	Y727C	Missense		
82	*PRKAR1A*		c.709(-7–2)del6				
	*PDE11A*		c.1142G>T	E382X	Codon stop		
83	*PRKAR1A*		c.279–282delTAGG				
	*PDE11A*		c.2180A>G	Y727C	Missense		
84	*PRKAR1A*		c.440 + 1G>A				
	*PDE11A*		c.171Tdel	T58PfsX41	Codon stop		
85	*PRKAR1A*		c.865 G>T				
	*PDE11A*		c.2180A>G	Y727C	Missense		
86	*PRKAR1A*		c.491–492delTG				
	*PDE11A*		c.2411G>A	R804H	Missense		
87	*PRKAR1A*		c.738T>G				
	*PDE11A*		c.824C>A	S275X	Codon stop		
88	*PRKAR1A*		c.353–365del13				
	*PDE11A*		c.2180A>G	Y727C	Missense		
89	*PRKAR1A*		c.709(-7–2)del6				
	*PDE11A*		c.652C>T	L218F	Missense		
90	*PRKAR1A*		c.502 + 1G>A				
	*PDE11A*		c.2180A>G	Y727C	Missense		
91	*PRKAR1A*		c.43–58del16				
	*PDE11A*		c.2632A>G	M878V	Missense		
92	*PRKAR1A*		c.491–492delTG				
	*PDE11A*		c.2180A>G	Y727C	Missense		
93	*PRKAR1A*		c.642dupT	p.Val215CysfsX18	Codon stop		Ali A. Ghazi (44), 2021
94	*PRKAR1A*	Exon 2	c.171–172insT	p.L57FfsX70			Safak Akin (45), 2017
95	*PRKAR1A*	Exon 2		C18G	Missense		Ran Hui (46), 2014
96	*PRKAR1A*	Exons 4A and 4B		S147N			Gu Yan-yun (47), 2004
97	*ARMC5*		c.1606C>G	p.P536A	Nonsense		Zhu Mingqiang (48), 2021
99	*PRKAR1A*		c.753 del AT				Patricia de Cremoux (49), 2008
100	*PRKAR1A*		c.846 ins A				
101	*PRKAR1A*		c.502C+1 G>T				
102	*PRKAR1A*		c.708C+1 G>T				
103	*PRKAR1A*		c.109 C>T				

The family genetic history was recorded in 30 cases; the pathogenic variants of 10 cases (10/30, 33.33%) were from their fathers, those of 13 cases (13/30, 43.33%) were from their mothers, and those of the remaining 7 cases were not from their parents. Among the 21 cases with mutated *PRKAR1A* gene, 11 (11/21, 52.38%) were inherited from the mothers and 6 (6/21, 28.57%) were from the fathers. Among the six cases with mutated *PDE11A* gene, three (3/6, 50%) were inherited from the mothers and two (2/6, 33.33%) were inherited from the fathers. The geographic distribution of gene mutation is shown in **Figure 2**.

**Figure 3:**
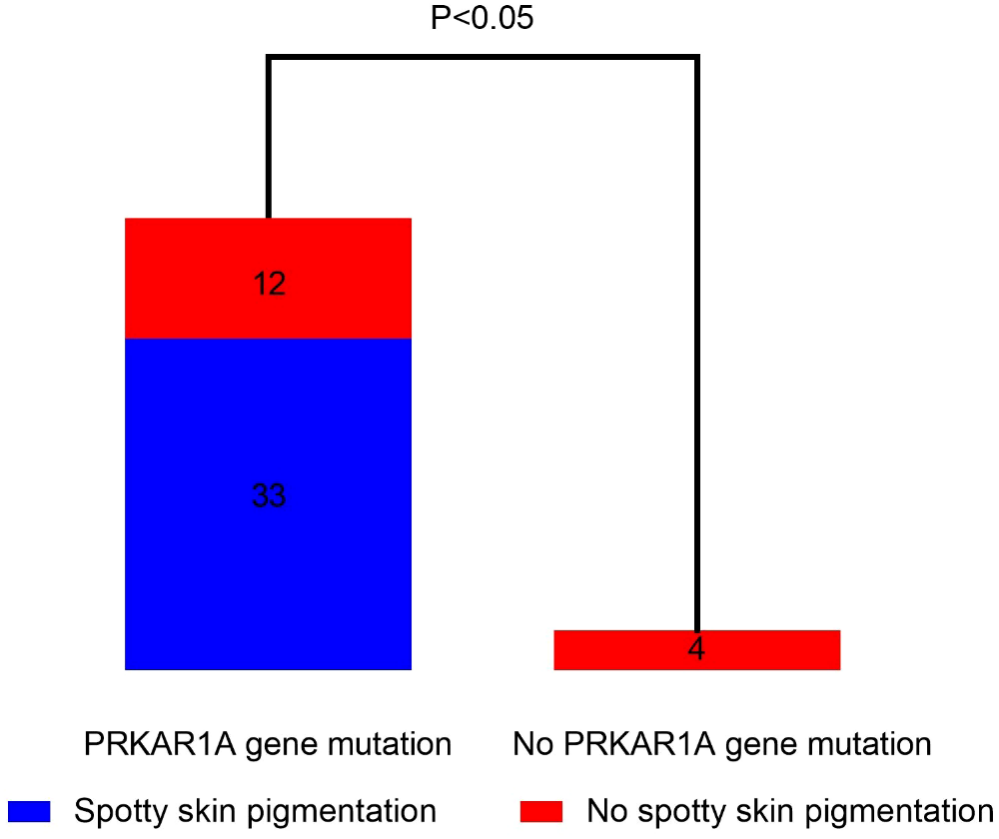
Correlation analysis of spotty skin pigmentation and PRKAR1A gene mutation in PPNAD patients with CNC.

The proportion of spotty skin pigmentation with a typical distribution in patients with cPPNAD with or without *PRKAR1A* gene mutation is presented in **Figure 3**. Spotty skin pigmentation was presented in 33 patients (33/45, 73.33%) with *PRKAR1A* gene mutation. There was no patient who presented with spotty skin pigmentation without *PRKAR1A* gene mutation. There was significant correlation between *PRKAR1A* gene mutation and spotty skin pigmentation with a typical distribution in cPPNAD (*p* < 0.05).

### 3.5 Treatment

The treatment regimens were recorded for 122 patients. A total of 62 patients (62/122, 50.82%) underwent bilateral adrenalectomy, 41 patients (41/122, 33.61%) underwent unilateral adrenalectomy, and 15 patients (15/122, 12.30%) underwent two-stage bilateral adrenalectomy (since unilateral adrenalectomy could not control the cortisol elevation, the other side of the adrenal gland was removed). Three patients (3/122, 2.46%) underwent unilateral total adrenalectomy and contralateral subtotal adrenalectomy, and one patient (1/122, 0.82%) underwent unilateral subtotal adrenalectomy.

Regarding the fertility status of four patients with PPNAD, notably, a 31-year-old woman with cPPNAD without adrenalectomy developed severe perinatal preeclampsia and gave birth to a baby girl with moderate respiratory distress syndrome (50). Moreover, a 28-year-old woman with CS secondary to cPPNAD had a successful pregnancy 3 years after bilateral adrenalectomy, but the perinatal period was not uneventful because of the need for hormone replacement (51). There were two women with PPNAD who had difficulty in pregnancy and gave birth to healthy babies after unilateral adrenalectomy with an uneventful perinatal period (31, 52).

Here are the following suggestions for choosing which side of the adrenal gland to remove. The side with more cortisol secretion with adrenal venous sampling (AVS) was selected (53, 54), the side with the larger volume with the use of 3D radiologic imaging volume analysis to compare the adrenal size was selected (55), the side with the highest uptake with 131-cholesterol removal radionuclide scan of adrenal gland was selected (56), and the side showing obvious unilateral adrenal nodules with CT or MRI scan was selected (56).

## 4 Discussion

The present study is the first systemic review to investigate the different clinical features and pathogenic variants of PPNAD in 23 countries on six continents. Our study showed that most of the patients with PPNAD were between 10 and 30 years old (71.88%), with a female-to-male ratio of 2:1. Of the patients with PPNAD, 94.29% had osteoporosis or osteopenia. Moreover, almost all patients lost the cortisol circadian rhythm (98.59%), and both plasma and urinary cortisol cannot be suppressed in all patients tested with both low-dose or high-dose dexamethasone and Liddle test (100%). In terms of genetic mutations, six different gene mutations, *PRKAR1A*, *PDE11A*, *PRKACA*, *CTNNB1*(*β-catenin*), *PDE8B*, and *ARMC5*, were identified. In particular, there was significant correlation between *PRKAR1A* gene mutation and spotty skin pigmentation with a typical distribution in cPPNAD (*p* < 0.05). More than half of the patients underwent bilateral adrenalectomy, and 33.61% of the patients underwent unilateral adrenalectomy.

Abnormal bone metabolism and growth retardation are more common in PPNAD compared to other causes of CS (57, 58). In our study, the age of patients with growth retardation was 1 to 19 years old, which may be secondary to early onset and prolonged exposure to hypercortisolemia, impairing growth and delaying skeletal maturation. Moreover, *PRKAR1A* gene mutation might lead to abnormal osteoblast differentiation (59–61). The main reason for the unsatisfactory height after treatment is the lack of catch-up growth. To solve this problem, children with PPNAD should be tested for GH and IGF-1 in time after surgery. If GH is insufficient, the application of human GH is encouraged. Early treatment and a longer remaining growth period contribute to the achievement of ideal adult height (62). There are also four patients with acromegaly due to GH-producing pituitary adenoma in our study, which together form CNC. Thus, patients with PPNAD should be highly suspected of pituitary adenoma if there is an increase in GH.

In our study, we found a total of six different gene mutations, *PRKAR1A*, *PRKACA*, *PDE11A*, *PDE8B*, *ARMC5*, and *CTNNB1* (*β-catenin*). Early studies had demonstrated that the development, proliferation, and function of adrenocortical cells are mainly regulated by the cyclic adenosine monophosphate protein kinase A (cAMP-PKA) pathway (63, 64). In particular, *CTNNB1* (*β-catenin*) gene mutation only occurs in the greater adrenal tubercle, but as a somatic mutation, it may also be involved in secondary tumors based on primary hyperplasia (34, 40). Although *PRKAR1A* gene mutation is a supplemental criterion for CNC diagnosis, the diagnosis of CNC cannot be ruled out if *PAKAR1A* gene mutation is not detected. Genetic testing results of *PRKAR1A*, *PRKACA*, or *PDE11A* mutations are helpful for the diagnosis of CNC (11). I-PPNAD may be closely related to C. 709(-7–2) del6 or *M1V* mutation of *PRKAR1A* (65–67). As a genetic modifying factor for the development of testicular and adrenal tumors in patients with germline *PRKAR1A* mutation, *PDE11A* is probably a phenotype modifying gene but not a causative gene. Therefore, *PDE11A* mutation might indicate the occurrence of PPNAD and other types of adrenal tumors (42, 68). *ARMC5* constitutional variant was reported “nonsense” in an Asian patient with PPNAD, but the P536A is a missense variant, and frequent especially in an Asian population (MAF = 0.002) (48). Some cases of corticotropin-independent macronodular adrenal hyperplasia often appear to be with inactivating mutations of *ARMC5* (69, 70). Therefore, patients with PPNAD with *ARMC5* gene mutation might combine with macronodular adrenal hyperplasia.

The study showed that the correlation between spotty skin pigmentation and *PRKAR1A* gene mutation was analyzed in patients with cPPNAD. The results showed that there was significant correlation between *PRKAR1A* gene mutation and spotty skin pigmentation in patients with cPPNAD. This is consistent with the findings of Jerome Bertherat et al. (71). PKA signaling promotes melanogenesis in melanocytes by phosphorylating CREB, which results in increased MITF transcription and subsequent increased expression of tyrosinase, TRP-1, and TRP-2. *PRKAR1A* deficiency produces large amounts of melanin and presents as darkly pigmented cutaneous papules or nodules (72). Therefore, the occurrence of spotty skin pigmentation in patients with CS should be highly suspicious of cPPNAD and *PAKAR1A* gene mutation.

Among all the patients included in this review, no mutated genes were reported in 19 cases. The reason is partly due to the limited gene sequence template selection, and the other is the somatic events. The tumor specimen might not obtain the corresponding tissue and was contaminated by the surrounding tissue. In case of somatic events, laser-captured micro-dissected cells can be used (36), and blood tissue can also be tested for genetic mutations to identify germline or somatic mutations.

Patients with PPNAD presented with an ACTH-independent form of CS, characterized by decreased or undetectable ACTH levels, elevated serum cortisol concentrations with loss of circadian rhythm, and paradoxical increase in UFC excretion after HDDST (Liddle test), with a maximum specificity of 100% (6, 39, 73, 74). Stratakis et al. found that all patients with PPNAD had a 100% or greater increase in urinary free cortisol excretion on day 6 of the Liddle test (6). The paradoxical rise in serum cortisol levels following Liddle’s test implied that glucocorticoids can locally regulate adrenocortical steroidogenesis in the majority of PPNAD. In at least some PPNAD tissues, aberrant coupling of the glucocorticoid receptor (GR) to the cAMP–PKA pathway instead of GR overexpression may be the culprit for the dexamethasone-induced rise in cortisol production (31). Different from the pathological features of isolated micronodular adrenocortical disease (i-MAD), PPNAD has small nodular staining and internodular atrophy, and the cortex was clearly segmented (75, 76). However, adrenal tissue acquisition was invasive, and a patient with a psychiatric disorder concealed a history of exogenous cortisol intake, while laboratory findings were consistent with PPNAD, leading physicians to misdiagnose and remove the normal adrenal gland (77). In the future, PPNAD gene mutation diagnosis due to blood might be the first choice for diagnosis and next-generation sequencing (NGS) should be preferred (22).

DHEA-S is a specific and stable marker of adrenal androgen secretion, which may also be a good predictor of risk for postoperative adrenal insufficiency, and ACTH plays an important role in its regulation (78, 79). Circulating DHEA-S levels are significantly reduced in patients with CS due to adrenocortical adenomas (78). However, in our study, patients with PPNAD can have a higher level of DHEA-S than normal, when combined with pituitary adenoma (21) or adrenal carcinoma (19). Therefore, if there is abnormal increase in DHEA-S, attention should be paid to whether it is complicated with other types of adrenal tumors and pituitary tumors.

Clinical management is also a complex issue to be discussed for patients with PPNAD. Xu et al. reported that 12 of 13 patients with PPNAD had clinical and laboratory remission of CS after unilateral adrenalectomy (56). Regarding the fertility status of four women with PPNAD, a woman without adrenalectomy developed severe perinatal preeclampsia and gave birth to a baby girl with moderate respiratory distress syndrome (50). Furthermore, a woman experienced a not uneventful perinatal period after bilateral adrenalectomy due to the need for hormone replacement (51). There were two women with PPNAD who gave birth to healthy babies after unilateral adrenalectomy with an uneventful perinatal period (31, 52). According to the included pregnant women, it is recommended that pre-pregnant women with PPNAD should undergo unilateral adrenalectomy to correct hypercortisolemia before pregnancy, and hydrocortisone can be given during delivery as a preventive measure (51, 52).

For patients who failed to effectively control elevated cortisol before or after adrenalectomy, ketoconazole, metyrapone, mitotane, and trilostane are effective in correcting hypercortisolemia through inhibition of steroidogenesis. Fluconazole has recently been proposed as a safer alternative to ketoconazole (80). After unilateral adrenalectomy, an individualized approach with close follow-up can lead to good clinical outcomes; dexamethasone stimulation test and adrenal MRI can be used for postoperative monitoring of PPNAD; if CS recurs during follow-up, contralateral adrenalectomy should be performed, followed by lifelong glucocorticoid therapy (56).

Among patients with cPPNAD, 28.79% were associated with cardiac or cutaneous myxoma, which is the most lethal manifestation of CNC and requires vigilant preoperative examination by a cardiologist and careful postoperative follow-up. Myxomas alter valve function through outflow obstruction and valve growth and pose an embolic threat to the brain and other organs (81). The incidence of embolism was 18% in the isolated atrial myxoma group and 40% in the recurrent myxoma group; CNC should be considered in all patients with cardiac myxoma; CNC is more common in patients with recurrent cardiac myxomas, and often involves two or more cavities. Testing for mutations in patients with isolated myxomas or multiple myxomas at atypical sites and screening for mutations in their immediate relatives may help establish an early diagnosis of the disease and implement appropriate clinical follow-up to detect recurrence in these patients (82).

PPNAD is the most common endocrine tumor in CNC, and in order to detect other complications of CNC in time, especially life-threatening cardiac myxoma, pediatric patients should have an annual follow-up examination, which should include echocardiography, thyroid ultrasound, and pituitary function examination, and twice a year if myxoma is found. Oral glucose tolerance, thyroid hormone release, and pituitary function tests can detect growth-hormone-producing pituitary adenomas early in children before clinical symptoms (such as acromegaly) appear. Adolescent patients should also be closely monitored for abnormal changes in growth rate and pubertal status caused by large cell calcifying Sertoli cell tumors (LCCSCT) (21, 83, 84). In addition, testicular ultrasound examination is recommended for men, and abdominal and pelvic ultrasound examination and breast imaging are recommended for female patients (1, 85). One patient had a cPPNAD with bilateral papillary thyroid carcinoma occurring 11 years apart. Thus, follow-up means decades (86).

Our study has several limitations. Firstly, because of the long incubation period of comorbidities in CNC, there may be bias in the diagnosis of CNC in some patients who do not yet have comorbidities. Secondly, the limited number of pregnant patients treated by unilateral adrenalectomy (n:2) is another limitation. Although the number of pregnant women included is small, this article included pregnant women with bilateral adrenalectomy, unilateral adrenalectomy, and no surgery, to comprehensively understand the conditions of the perinatal. Thirdly, when analyzing the characteristics of patients with PPNAD, there are cases with insufficient clinical information, which prevented us from analyzing the characteristics of some rare mutations. Furthermore, there may be a selection bias in this study because many patients with PPNAD did not undergo genetic testing. The mechanisms by which different mutations lead to different clinical features and whether the mutated gene detected is the pathogenic gene of PPNAD remain unclear. Further studies are needed to explain the molecular mechanism of PPNAD more precisely.

Our systematic review, despite the above limitations, is, to our knowledge, the most comprehensive review on patients with PPNAD published to date and provides clinicians with vital information on the common presentation features that may help with the diagnosis and highlight management options. In particular, our study analyzed special groups of pregnant women and children, summarized treatment options and prognosis from the included patients, and summarized treatment options for these groups. Moreover, laboratory tests were also analyzed in detail, and patient tests for PPNAD with other endocrine tumors were analyzed. This study contributes to a comprehensive understanding of PPNAD, including clinical manifestations, laboratory findings, treatment, prognosis, and follow-up.

In conclusion, our study is the first systematic review to investigate the different clinical features, pathogenic variants, and treatments of PPNAD. For the young patients with CS, especially female patients with growth retardation, spotty skin pigmentation, and osteoporosis/low bone mineral density, PPNAD should be considered. For treatment, unilateral adrenalectomy is recommended, especially in women who are preparing for pregnancy. In view of the trauma and difficulty of pathology in PPNAD diagnosis, genetic testing before surgery might be a reasonable option. Patients with PPNAD with spotty skin pigmentation should consider *PRKAR1A* gene mutation and pay attention to CNC. In the future, both short- and long-term evaluations of the complications of PPNAD need to be carried out.

## Data availability statement

The raw data supporting the conclusions of this article will be made available by the authors, without undue reservation.

## Author contributions

JS: Conceptualization, Data curation, Writing – original draft, Writing – review & editing. LD: Conceptualization, Data curation, Writing – original draft, Writing – review & editing. LH: Data curation, Writing – original draft. HF: Data curation, Writing – original draft. RL: Writing – original draft. JF: Writing – original draft. JD: Writing – review & editing. LL: Writing – original draft, Writing – review & editing.

## References

[1] Stratakis CA Kirschner LS Carney JA . Clinical and molecular features of the Carney complex: diagnostic criteria and recommendations for patient evaluation. J Clin Endocrinol Metab. (2001) 86:4041–6. doi: 10.1210/jcem.86.9.7903 11549623

[2] Cazabat L Ragazzon B Groussin L Bertherat J . PRKAR1A mutations in primary pigmented nodular adrenocortical disease. Pituitary. (2006) 9(3):211–9. doi: 10.1007/s11102-006-0266-1 17036196

[3] Navarro Moreno C Delestienne A Marbaix E Aydin S Hörtnagel K Lechner S . Familial forms of Cushing syndrome in primary pigmented nodular adrenocortical disease presenting with short stature and insidious symptoms: A clinical series. Horm Res Paediatr. (2018) 89(6):423–33. doi: 10.1159/000488761 29909407

[4] Jarial KDS Walia R Nahar U Bhansali A . Primary bilateral adrenal nodular disease with Cushing's syndrome: varying aetiology. BMJ Case Rep. (2017) 2017:bcr-2017-220154. doi: 10.1136/bcr-2017-220154 PMC5747626 28739615

[5] Mete O Erickson LA Juhlin CC de Krijger RR Sasano H Volante M . Overview of the 2022 WHO classification of adrenal cortical tumors. Endocr Pathol. (2022) 33(1):155–96. doi: 10.1007/s12022-022-09710-8 PMC8920443 35288842

[6] Stratakis CA Sarlis N Kirschner LS Carney JA Doppman JL Nieman LK . Paradoxical response to dexamethasone in the diagnosis of primary pigmented nodular adrenocortical disease. Ann Intern Med. (1999) 131(8):585–91. doi: 10.7326/0003-4819-131-8-199910190-00006 10523219

[7] George J Vimal MV Bandgar T Menon PS Shah NS . An unusual variant of Cushing syndrome. Endocr Pract. (2008) 14:717–20. doi: 10.4158/EP.14.6.717 18996791

[8] Xu YY Li YY Chen QL Ma HM Zhang J Guo S . [A case of primary pigmented nodular adrenocortical disease caused by somatic variation of the PRKACA gene]. Zhonghua Er Ke Za Zhi. (2023) 61(1):76–8. doi: 10.3760/cma.j.cn112140-20220626-00589 36594126

[9] Wan S Zhang TT Chen T Zhang D Mo D Xu J . Primary pigmented nodular adrenal disease: a report of three cases. Zhonghua nei ke za zhi. (2022) 61(8):944–7. doi: 10.3760/cma.j.cn112138-20211031-00760 35922222

[10] Tsurutani Y Kiriyama K Kondo M Hasebe M Sata A Mizuno Y . Carney Complex Complicated with Primary Pigmented Nodular Adrenocortical Disease without Cushing's Syndrome Recurrence for Five Years after Unilateral Adrenalectomy. Intern Med. (2022) 61(2):205–11. doi: 10.2169/internalmedicine.7418-21 PMC8851166 35034934

[11] Sun Q Song J Feng W Wang C Yang X Zhang M . Carney complex presenting as subclinical Cushing syndrome in a child due to a novel Phosphodiesterase 11A mutation. Heliyon. (2022) 8(12):e12077. doi: 10.1016/j.heliyon.2022.e12077 36536910 PMC9758402

[12] Ferreira SH Costa MM Rios E Santos Silva R Costa C Castro-Correia C . Carney complex due to a novel pathogenic variant in the PRKAR1A gene - a case report. J Pediatr Endocrinol Metab. (2019) 32(2):197–202. doi: 10.1515/jpem-2018-0199 30699069

[13] Kiriakopoulos A Linos D . Carney syndrome presented as a pathological spine fracture in a 35-year-old male. Am J Case Rep. (2018) 19(8):1366–9. doi: 10.12659/AJCR.911962 PMC6251001 30442879

[14] Fu J Lai F Chen Y Wan X Wei G Li Y . A novel splice site mutation of the PRKAR1A gene, C.440+5 G>C, in a Chinese family with Carney complex. J Endocrinol Invest. (2018) 41(8):909–17. doi: 10.1007/s40618-017-0817-5 29318463

[15] Lan L Zhang G Deng W Wang H Ye L Ning G . To report and analyze the gene mutation in a family with primary pigmented nodular adrenocortical disease. Shandong Med J. (2017) 57(26):86–8. doi: 10.3969/j.issn.1002-266X.2017.26.029.

[16] Hernández-Ramírez LC Tatsi C Lodish MB Faucz FR Pankratz N Chittiboina P . Corticotropinoma as a component of carney complex. J Endocr Soc. (2017) 1(7):918–25. doi: 10.1210/js.2017-00231 PMC5686778 29264542

[17] Vezzosi D Tenenbaum F Cazabat L Tissier F Bienvenu M Carrasco CA . Hormonal, radiological, NP-59 scintigraphy, and pathological correlations in patients with Cushing's syndrome due to primary pigmented nodular adrenocortical disease (PPNAD). J Clin Endocrinol Metab. (2015) 100(11):4332–8. doi: 10.1210/jc.2015-2174 26390100

[18] Tung SC Hwang DY Yang JW Chen WJ Lee CT . An unusual presentation of Carney complex with diffuse primary pigmented nodular adrenocortical disease on one adrenal gland and a nonpigmented adrenocortical adenoma and focal primary pigmented nodular adrenocortical disease on the other. Endocr J. (2012) 59(9):823–30. doi: 10.1507/endocrj.EJ12-0040 22785148

[19] Morin E Mete O Wasserman JD Joshua AM Asa SL Ezzat S . Carney complex with adrenal cortical carcinoma. J Clin Endocrinol Metab. (2012) 97(2):E202–6. doi: 10.1210/jc.2011-2321 22112809

[20] Storr HL Metherell LA Dias R Savage MO Rasmussen AK Clark AJ . Familial isolated primary pigmented nodular adrenocortical disease associated with a novel low penetrance PRKAR1A gene splice site mutation. Horm Res Paediatr. (2010) 73(2):115–9. doi: 10.1159/000277629 20190548

[21] Urban C Weinhäusel A Fritsch P Sovinz P Weinhandl G Lackner H . Primary pigmented nodular adrenocortical disease (PPNAD) and pituitary adenoma in a boy with sporadic carney complex due to a novel, de novo paternal PRKAR1A mutation (R96X). J Pediatr Endocrinol Metab. (2007) 20(2):247–52. doi: 10.1515/JPEM.2007.20.2.247 17396442

[22] Kamilaris CDC Faucz FR Andriessen VC Nilubol N Lee CR Ahlman MA . First somatic PRKAR1A defect associated with mosaicism for another PRKAR1A mutation in a patient with Cushing syndrome. J Endocr Soc. (2021) 5(4):bvab007. doi: 10.1210/jendso/bvab007 33644619 PMC7885549

[23] Zhang CD Pichurin PN Bobr A Lyden ML Young WF Bancos I . Cushing syndrome: uncovering Carney complex due to novel PRKAR1A mutation. Endocrinol Diabetes Metab Case Rep. (2019) 2019:18–0150. doi: 10.1530/EDM-18-0150 PMC6432981 30897549

[24] Kyrilli A Lytrivi M Bouquegneau MS Demetter P Lucidi V Garcia C . Unilateral adrenalectomy could be a valid option for primary nodular adrenal disease: evidence from twins. J Endocr Soc. (2018) 3(1):129–34. doi: 10.1210/js.2018-00261 PMC6302904 30591956

[25] Tirosh A Lodish MB Lyssikatos C Belyavskaya E Papadakis GZ Stratakis CA . Circadian plasma cortisol measurements reflect severity of hypercortisolemia in children with different etiologies of endogenous Cushing syndrome. Horm Res Paediatr. (2017) 87(5):295–300. doi: 10.1159/000464463 28433999 PMC5506540

[26] Pasternak-Pietrzak K Stratakis CA Moszczyńska E Lecka-Ambroziak A Staniszewski M Watrobińska U . Detection of new potentially pathogenic mutations in two patients with primary pigmented nodular adrenocortical disease (PPNAD) - case reports with literature review. Endokrynol Pol. (2018) 69(6):675–81. doi: 10.5603/EP.a2018.0063 PMC6347113 30259502

[27] Korpaisarn S Trachoo O Panthan B Aroonroch R Suvikapakornkul R Sriphrapradang C . A novel PRKAR1A mutation identified in a patient with isolated primary pigmented nodular adrenocortical disease. Case Rep Oncol. (2017) 10(2):769–76. doi: 10.1159/000479585 PMC5582444 28878664

[28] Mineo R Tamba S Yamada Y Okita T Kawachi Y Mori R . A Novel Mutation in the type Iα Regulatory Subunit of Protein Kinase A (PRKAR1A) in a Cushing's Syndrome Patient with Primary Pigmented Nodular Adrenocortical Disease. Intern Med. (2016) 55(17):2433–8. doi: 10.2169/internalmedicine.55.6605 27580546

[29] da Silva RM Pinto E Goldman SM Andreoni C Vieira TC Abucham J . Children with Cushing's syndrome: Primary Pigmented Nodular Adrenocortical Disease should always be suspected. Pituitary. (2011) 14(1):61–7. doi: 10.1007/s11102-010-0260-5 20924687

[30] Poukoulidou T Maiter D Bertherat J Beauloye V . A rare case of familial Cushing's syndrome with a common presentation of weight gain due to a mutation of the PRKAR1A gene causing isolated primary pigmented nodular adrenocortical disease. J Pediatr Endocrinol Metab. (2014) 27(9-10):1005–9. doi: 10.1515/jpem-2014-0018 24859511

[31] Hofland J de Herder WW Derks L Hofland LJ van Koetsveld PM de Krijger RR . Regulation of steroidogenesis in a primary pigmented nodular adrenocortical disease-associated adenoma leading to virilization and subclinical Cushing's syndrome. Eur J Endocrinol. (2012) 168(1):67–74. doi: 10.1530/EJE-12-0594 23065993 PMC4100689

[32] Anselmo J Medeiros S Carneiro V Greene E Levy I Nesterova M . A large family with carney complex caused by the S147G PRKAR1A mutation shows a unique spectrum of disease including adrenocortical cancer. J Clin Endocrinol Metab. (2012) 97(2):351–9. doi: 10.1210/jc.2011-2244 PMC3275364 22112814

[33] Peck MC Visser BC Norton JA Pasche L Katznelson L . A novel PRKAR1A mutation associated with primary pigmented nodular adrenocortical disease and the Carney complex. Endocr Pract. (2010) 16(2):198–204. doi: 10.4158/EP09245 19833579

[34] Tadjine M Lampron A Ouadi L Horvath A Stratakis CA Bourdeau I . Detection of somatic beta-catenin mutations in primary pigmented nodular adrenocortical disease (PPNAD). Clin Endocrinol (Oxf). (2008) 69(3):367–73. doi: 10.1111/j.1365-2265.2008.03273.x PMC3138207 18419788

[35] Bandelin PB Moreno AJ Lemar HJ Stratakis CA Oliver TG . The use of positron emission tomography-computed tomography scan in the evaluation of a patient with carney complex. J Clin Endocrinol Metab. (2008) 93(8):2946–7. doi: 10.1210/jc.2008-0313 PMC2515079 18685116

[36] Almeida MQ Brito LP Domenice S Costa MH Pinto EM Osório CA . Absence of PRKAR1A loss of heterozygosity in laser-captured microdissected pigmented nodular adrenocortical tissue from a patient with carney complex caused by the novel nonsense mutation p.Y21X. Arq Bras Endocrinol Metabol. (2008) 52(8):1257–63. doi: 10.1590/S0004-27302008000800009 19169478

[37] Horvath A Mericq V Stratakis CA . Mutation in PDE8B, a cyclic AMP-specific phosphodiesterase in adrenal hyperplasia. N Engl J Med. (2008) 358:750–2. doi: 10.1056/NEJMc0706182 18272904

[38] Horvath A Giatzakis C Robinson-White A Boikos S Levine E Griffin K . Adrenal hyperplasia and adenomas are associated with inhibition of phosphodiesterase 11A in carriers of PDE11A sequence variants that are frequent in the population. Cancer Res. (2006) 66(24):11571–5. doi: 10.1158/0008-5472.CAN-06-2914 17178847

[39] Bourdeau I Lacroix A Schürch W Caron P Antakly T Stratakis CA . Primary Pigmented nodular adrenocortical disease: Paradoxical responses of cortisol secretion to dexamethasone occur in vitro and are associated with increased expression of the glucocorticoid receptor. J Clin Endocrinol Metab. (2003) 88(8):3931–7. doi: 10.1210/jc.2002-022001 12915689

[40] Groussin L Jullian E Perlemoine K Louvel A Leheup B Luton JP . Mutations of the PRKAR1A gene in Cushing's syndrome due to sporadic primary pigmented nodular adrenocortical disease. J Clin Endocrinol Metab. (2002) 87(9):4324–9. doi: 10.1210/jc.2002-020592 12213893

[41] Kubo H Tsurutani Y Sugisawa C Sunouchi T Hirose R Saito J . Phenotypic variability in a family with carney complex accompanied by a novel mutation involving PRKAR1A. Tohoku J Exp Med. (2022) 257(4):337–45. doi: 10.1620/tjem.2022.J051 35732416

[42] Carney JA Gaillard RC Bertherat J Stratakis CA . Familial micronodular adrenocortical disease, Cushing syndrome, and mutations of the gene encoding phosphodiesterase 11A4 (PDE11A). Am J Surg Pathol. (2010) 34(4):547–55. doi: 10.1097/PAS.0b013e3181d31f49 PMC4042182 20351491

[43] Libé R Horvath A Fratticci A Vezzosi D Coste J Guillaud-Bataille M . ESE Young Investigator Award Frequent phosphodiesterase 11a (pde11a4) gene mutations in patients with carney complex (cnc) due to prkar1a mutations and adrenal (ppnad) and Sertoli cell tumors (lccsct): A digenic disorder? Endocrine Abstracts. (2010) 22:H1.3. doi: 10.1210/jc.2010-1704

[44] Ghazi AA Mandegar MH Abazari M Behzadnia N Sadeghian T Torbaghan SS . A novel mutation in PRKAR1A gene in a patient with Carney complex presenting with pituitary macroadenoma, acromegaly, Cushing's syndrome and recurrent atrial myxoma. Arch Endocrinol Metab. (2021) 65(3):376–80. doi: 10.20945/2359-3997000000369 PMC10065344 33939912

[45] Akin S Noyan S Dagdelen S Pasaoglu I Kaynaroglu V Askun MM . Unusual presentations of Carney Complex in patient with a novel PRKAR1A mutation. Neuroendocrinol Lett. (2017) 38(4):248–54. 28871709

[46] Ran H Ma X Wang Q Xie Z Ding Y Qin G . A pedigree study of a patient with primary pigmented nodular adrenocortical disease and familial gene mutation. Zhonghua nei ke za zhi. (2014) 53(5):398–402. doi: 10.3760/cma.j.issn.0578-1426.2014.05.015 25146409

[47] Gu Y Chen Y Song H Li X Luo T Qiao J . Clinical and molecular research in a case of familial Carney complex. Zhonghua nei ke za zhi. (2004) 43:764–8. 15631831

[48] Zhu M Dong G Huang K Chen X Zhang L Dai Y . Central precocious puberty with primary pigmented nodular adrenocortical disease:One case report. Chin J Endocrinol Metab. (2021) 37:240–4. doi: 10.3760/cma.j.cn311282-20200212-00057

[49] de Cremoux P Rosenberg D Goussard J Brémont-Weil C Tissier F Tran-Perennou C . Expression of progesterone and estradiol receptors in normal adrenal cortex, adrenocortical tumors, and primary pigmented nodular adrenocortical disease. Endocrine-Related Cancer. (2008) 15(2):465–74. doi: 10.1677/ERC-07-0081 18508999

[50] Schulz S Redlich A Köppe I Reschke K Weise W . Carney complex - An unexpected finding during puerperium. Gynecol Obstet Invest. (2001) 51(3):211–3. doi: 10.1159/000052927 11306912

[51] Kalelioglu I Mert M Has R Kale T Iyibozkurt C Aral F . Carney's complex: A successful pregnancy after bilateral adrenalectomy. Arch Med Sci. (2012) 8(1):175–7. doi: 10.5114/aoms.2012.27300 PMC3309456 22457694

[52] Cohen O Bogat S Dolitzki M Karasik A . Successful pregnancy after unilateral adrenalectomy in a case of primary pigmented adrenocortical disease. J Matern Fetal Neonatal Med. (2005) 17(2):161–3. doi: 10.1080/14767050500043210 16076627

[53] Peng X Yu Y Ding Y Yang F Chen X Chang C . Adrenal venous sampling as used in a patient with primary pigmented nodular adrenocortical disease. Transl Cancer Res. (2017) 6(6):1117–22. doi: 10.21037/tcr.2017.12.03

[54] Hackman KL Davis AL Curnow PA Serpell JW McLean CA Topliss DJ . Cushing syndrome in a young woman due to primary pigmented nodular adrenal disease. Endocr Pract. (2010) 16(1):84–8. doi: 10.4158/EP09177.CR 19703806

[55] Chrysostomou PP Lodish MB Turkbey EB Papadakis GZ Stratakis CA . Use of 3-dimensional volumetric modeling of adrenal gland size in patients with primary pigmented nodular adrenocortical disease. Horm Metab Res. (2016) 48(4):242–6. doi: 10.1055/s-0042-103686 PMC6300994 27065461

[56] Xu Y Rui W Qi Y Zhang C Zhao J Wang X . The role of unilateral adrenalectomy in corticotropin-independent bilateral adrenocortical hyperplasias. World J Surg. (2013) 37(7):1626–32. doi: 10.1007/s00268-013-2059-9 23592061

[57] Chen S Li R Lu L Duan L Zhang X Tong A . Efficacy of dexamethasone suppression test during the diagnosis of primary pigmented nodular adrenocortical disease in Chinese adrenocorticotropic hormone-independent Cushing syndrome. Endocrine. (2018) 59(1):183–90. doi: 10.1007/s12020-017-1436-9 PMC5765188 29094256

[58] Zhou J Zhang M Bai X Cui S Pang C Lu L . Demographic characteristics, etiology, and comorbidities of patients with Cushing's syndrome: A 10-year retrospective study at a large general hospital in China. Int J Endocrinol. (2019) 2019:7159696. doi: 10.1155/2019/7159696 30915114 PMC6399544

[59] Savage MO Scommegna S Carroll PV Ho JT Monson JP Besser GM . Growth in disorders of adrenal hyperfunction. Horm Res. (2002) 58 Suppl 1:39–43. doi: 10.1159/000064767 12373013

[60] Magiakou MA Mastorakos G Chrousos GP . Final stature in patients with endogenous Cushing's syndrome. J Clin Endocrinol Metab. (1994) 79:1082–5. doi: 10.1210/jcem.79.4.7962277 7962277

[61] Maillet M Bourdeau I Lacroix A . Update on primary micronodular bilateral adrenocortical diseases. Curr Opin Endocrinol Diabetes Obes. (2020) 27:132–9. doi: 10.1097/MED.0000000000000538 32209819

[62] Lebrethon MC Grossman AB Afshar F Plowman PN Besser GM Savage MO . Linear growth and final height after treatment for Cushing's disease in childhood. J Clin Endocrinol Metab. (2000) 85(9):3262–5. doi: 10.1210/jcem.85.9.6817 10999819

[63] Lodish M Stratakis CA . A genetic and molecular update on adrenocortical causes of Cushing syndrome. Nat Rev Endocrinol. (2016) 12:255–62. doi: 10.1038/nrendo.2016.24 26965378

[64] Mazzuco TL Durand J Chapman A Crespigio J Bourdeau I . Genetic aspects of adrenocortical tumours and hyperplasias. Clin Endocrinol (Oxf). (2012) 77(1):1–10. doi: 10.1111/j.1365-2265.2012.04403.x 22471738

[65] Horvath A Bertherat J Groussin L Guillaud-Bataille M Tsang K Cazabat L . Mutations and polymorphisms in the gene encoding regulatory subunit type 1-alpha of protein kinase A (PRKAR1A): an update. Hum Mutat. (2010) 31(4):369–79. doi: 10.1002/humu.21178 PMC2936101 20358582

[66] Pereira AM Hes FJ Horvath A Woortman S Greene E Bimpaki E . Association of the M1V PRKAR1A mutation with primary pigmented nodular adrenocortical disease in two large families. J Clin Endocrinol Metab. (2010) 95(1):338–42. doi: 10.1210/jc.2009-0993 PMC2805491 19915019

[67] Groussin L Horvath A Jullian E Boikos S Rene-Corail F Lefebvre H . A PRKAR1A mutation associated with primary pigmented nodular adrenocortical disease in 12 kindreds. J Clin Endocrinol Metab. (2006) 91(5):1943–9. doi: 10.1210/jc.2005-2708 16464939

[68] Libé R Horvath A Vezzosi D Fratticci A Coste J Perlemoine K . Frequent phosphodiesterase 11A gene (PDE11A) defects in patients with carney complex (CNC) caused by PRKAR1A mutations: PDE11A may contribute to adrenal and testicular tumors in CNC as a modifier of the phenotype. J Clin Endocrinol Metab. (2011) 96(1):E208–14. doi: 10.1210/jc.2010-1704 PMC3038483 21047926

[69] Assié G Libé R Espiard S Rizk-Rabin M Guimier A Luscap W . ARMC5 mutations in macronodular adrenal hyperplasia with Cushing's syndrome. N Engl J Med. (2013) 369(22):2105–14. doi: 10.1056/NEJMoa1304603 PMC4727443 24283224

[70] Faucz FR Zilbermint M Lodish MB Szarek E Trivellin G Sinaii N . Macronodular adrenal hyperplasia due to mutations in an armadillo repeat containing 5 (ARMC5) gene: a clinical and genetic investigation. J Clin Endocrinol Metab. (2014) 99(6):E1113–9. doi: 10.1210/jc.2013-4280 PMC4037724 24601692

[71] Bertherat J Horvath A Groussin L Grabar S Boikos S Cazabat L . Mutations in regulatory subunit type 1A of cyclic adenosine 5′-monophosphate-dependent protein kinase (PRKAR1A): Phenotype analysis in 353 patients and 80 different genotypes. J Clin Endocrinol Metab. (2009) 94(6):2085–91. doi: 10.1210/jc.2008-2333 PMC2690418 19293268

[72] Cohen JN Joseph NM North JP Onodera C Zembowicz A LeBoit PE . Genomic analysis of pigmented epithelioid melanocytomas reveals recurrent alterations in PRKAR1A, and PRKCA genes. Am J Surg Pathol. (2017) 41(10):1333–46. doi: 10.1097/PAS.0000000000000902 28796000

[73] Louiset E Stratakis CA Perraudin V Griffin KJ Libé R Cabrol S . The paradoxical increase in cortisol secretion induced by dexamethasone in primary pigmented nodular adrenocortical disease involves a glucocorticoid receptor-mediated effect of dexamethasone on protein kinase A catalytic subunits. J Clin Endocrinol Metab. (2009) 94(7):2406–13. doi: 10.1210/jc.2009-0031 PMC2708955 19383776

[74] Tizianel I Barbot M Ceccato F . Subtyping of Cushing's syndrome: a step ahead. Exp Clin Endocrinol Diabetes. (2024). doi: 10.1055/a-2299-5065 38574761

[75] Shenoy BV Carpenter PC Carney JA . Bilateral primary pigmented nodular adrenocortical disease. Rare cause of the Cushing syndrome. Am J Surg Pathol. (1984) 8:335–44. doi: 10.1097/00000478-198405000-00002 6329005

[76] Stratakis CA Boikos SA . Genetics of adrenal tumors associated with Cushing's syndrome: a new classification for bilateral adrenocortical hyperplasias. Nat Clin Pract Endocrinol Metab. (2007) 3:748–57. doi: 10.1038/ncpendmet0648 17955016

[77] Pineyro MM Redes L De Mattos S Sanchez L Brignardello E Bianchi V . Factitious Cushing's syndrome: A diagnosis to consider when evaluating hypercortisolism. Front Endocrinol (Lausanne). (2019) 10:129. doi: 10.3389/fendo.2019.00129 30886602 PMC6409302

[78] Yamaji T Ishibashi M Sekihara H Itabashi A Yanaihara T . Serum dehydroepiandrosterone sulfate in Cushing's syndrome. J Clin Endocrinol Metab. (1984) 59(6):1164–8. doi: 10.1210/jcem-59-6-1164 6238041

[79] Morio H Terano T Yamamoto K Tomizuka T Oeda T Saito Y . Serum levels of dehydroepiandrosterone sulfate in patients with asymptomatic cortisol producing adrenal adenoma: comparison with adrenal Cushing's syndrome and non-functional adrenal tumor. Endocr J. (1996) 43(4):387–96. doi: 10.1507/endocrj.43.387 8930526

[80] Riedl M Maier C Zettinig G Nowotny P Schima W Luger A . Long term control of hypercortisolism with fluconazole: case report and in vitro studies. Eur J Endocrinol. (2006) 154(4):519–24. doi: 10.1530/eje.1.02120 16556713

[81] Rothschild JA Kreso M Slodzinski M . Sudden death in a patient with carney's complex. Anesth Pain Med. (2013) 2:182–5. doi: 10.5812/aapm PMC3821135 24223358

[82] Vargas-Barrón J Vargas-Alarcón G Roldán F Vázquez-Antona C Vásquez Ortiz Z Erdmenger-Orellana J . Cardiac Myxomas and the Carney Complex. Revista Española de Cardiolog a (English Edition) (2008) 61(11):1205–9. doi: 10.1157/13127852 19000496

[83] Bertherat J . Carney complex (CNC). Orphanet J Rare Dis. (2006) 1:21. doi: 10.1186/1750-1172-1-21 16756677 PMC1513551

[84] Espiard S Vantyghem MC Assié G Cardot-Bauters C Raverot G Brucker-Davis F . Frequency and incidence of carney complex manifestations: A prospective multicenter study with a three-year follow-up. J Clin Endocrinol Metab. (2020) 105(3):dgaa002. doi: 10.1210/clinem/dgaa002 31912137

[85] Stratakis CA Ball DW . A concise genetic and clinical guide to multiple endocrine neoplasias and related syndromes. J Pediatr Endocrinol Metab. (2000) 13:457–65. doi: 10.1515/JPEM.2000.13.5.457 10803862

[86] Tung S Wang P Huang T Yang J Chen W . Carney complex with primary pigmented nodular adrenocortical disease and bilateral papillary thyroid carcinoma occurring 11 years apart - A case report. Endocrinologist. (2005) 15:243–7. doi: 10.1097/01.ten.0000170845.50695.27

